# Tceal7 Regulates Skeletal Muscle Development through Its Interaction with Cdk1

**DOI:** 10.3390/ijms24076264

**Published:** 2023-03-27

**Authors:** Zhenzhen Xiong, Mengni Wang, Jianhua Wu, Xiaozhong Shi

**Affiliations:** School of Biology and Biological Engineering, South China University of Technology, Guangzhou 510006, China

**Keywords:** Tceal7, Cdk1, skeletal muscle development

## Abstract

We have previously reported *Tceal7* as a muscle-specific gene that represses myoblast proliferation and promotes myogenic differentiation. The regulatory mechanism of *Tceal7* gene expression has been well clarified recently. However, the underlying mechanism of Tceal7 function in skeletal muscle development remains to be elucidated. In the present study, we have generated an MCK 6.5 kb-HA-Tceal7 transgenic model. The transgenic mice are born normally, while they have displayed defects in the growth of body weight and skeletal muscle myofiber during postnatal development. Although four RxL motifs have been identified in the Tceal7 protein sequence, we have not detected any direct protein-protein interaction between Tceal7 and Cyclin A2, Cyclin B1, Cylin D1, or Cyclin E1. Further analysis has revealed the interaction between Tceal7 and Cdk1 instead of Cdk2, Cdk4, or Cdk6. Transgenic overexpression of Tceal7 reduces phosphorylation of 4E-BP1 Ser65, p70S6K1 Thr389, and Cdk substrates in skeletal muscle. In summary, these studies have revealed a novel mechanism of Tceal7 in skeletal muscle development.

## 1. Introduction

The cell cycle progression is driven by the dimeric complexes of Cyclin and Cyclin-dependent kinases (Cdks), which specifically recognize and phosphorylate the serine/threonine-proline (S/T-P) or serine/threonine-proline-x-arginine/lysine (S/T-P-X-R/K) motif [1,2,3,4]. Overall, these Cyclin/Cdk complexes are activated orderly and function ordinately to guide the cell cycle [1]. Cyclin D/Cdk4(Cdk6) is activated first in the early G1 phase; Cyclin E/Cdk2 is then activated in the late G1 phase to ensure entry into the S phase. Cyclin A/Cdk2 is activated and coordinates DNA replication in the S phase, followed by the activation of Cyclin A/Cdk1, which is in charge of G2 phase progression [5]. Cyclin B-Cdk1 promotes the completion of cell division during the mitotic phase (M) [1,5]. Reducing the activity of Cdk1 slows down the overall translation rate in transformed cells as well as non-transformed primary mouse embryonic fibroblasts [6]. Further in vivo studies have demonstrated that Cdk1 is the essential Cdk required in early embryo development [7]. Cdk1 is sufficient to drive mouse embryos to undergo organogenesis and develop to gestation, while complete deletion of Cdk1 prevents mouse embryos from developing to morula and blastocyst stages [7]. A recent study has also reported that Cdk1 plays an essential role in muscle fiber growth upon muscle overload [8]. Cdk1 promotes the initiation and elongation procedures of protein synthesis through multiple pathways, such as eIF2α, 4EBP and p70S6K1 signaling [6,9].

Both 4E-BP1 and p70S6K1 are regulated by stepwise phosphorylation to initiate protein synthesis [10]. Non-phosphorylated 4E-BP1 has a negative role in cap-dependent translation initiation through its association with eIF4E, thereby interfering with the protein-protein interaction between eIF4E and eIF4G. Phosphorylation of 4E-BP1 disrupts its interaction with eIF4E and then releases its inhibitory effect on the assembly of the translation initiation complex [11]. There are seven phosphorylation sites in 4E-BP1: Thr37, Thr46, Ser65, Thr70, Ser83, Ser101, and Ser112 [12]. The phosphorylation of Thr37 and Thr46 is the priming phosphorylation, followed by the phosphorylation of Thr70 and finally, the phosphorylation of Ser65 [13,14]. Although phosphorylation of Thr37 and Thr46 fails to dissociate 4E-BP1 from eIF4E, it is essential to trigger subsequent phosphorylation of 4E-BP1 Thr70 and Ser65 [15]. p70S6K1 is activated by sequential phosphorylation events to regulate the translation of TOP mRNA by its substrates, such as eEF2, eEF1A and some ribosomal proteins [16,17]. Initial phosphorylation at Ser411, Ser418, Thr421 and Ser424 serves to release p70S6K1 from autoinhibition [18,19]. Phosphorylation of Thr389 results in particular activation of p70S6K1, while further PDK1-mediated phosphorylation of Thr229 is required for its full activity in vivo [17,20,21]. An alternative mechanism of p70S6K1 activation has also been reported [17].

We have previously identified a striated muscle-specific gene, Tceal7 [22]. Our recent work has elucidated a regulatory mechanism of its interesting expression pattern during skeletal muscle regeneration [23]. However, the in vivo function of Tceal7 is still mysterious, although that overexpression of Tceal7 in C2C12 cells results in slower cell cycle progression and enhanced myogenic differentiation [22]. In the present study, we have further investigated the underlying mechanism of Tceal7 in skeletal muscle development utilizing MCK 6.5 kb-HA-Tceal7 transgenic model. Interestingly, we have observed that transgenic mice are smaller than their wild-type littermates in body size during postnatal development and that H&E staining of skeletal muscle of transgenic mice is similar to that of wild-type controls, while the cross-sectional area of skeletal muscle fiber is significantly reduced. In addition, we have shown that Tceal7 directly interacts with Cdk1 and that transgenic overexpression of Tceal7 reduces the phosphorylation of 4E-BP1, p70S6K1, and Cdk substrates. In summary, our data support the notion that Tceal7 is an important regulator in skeletal muscle development.

## 2. Results

### 2.1. Construction of MCK 6.5 kb-HA-Tceal7 Transgenic Lines

**Figure 1:**
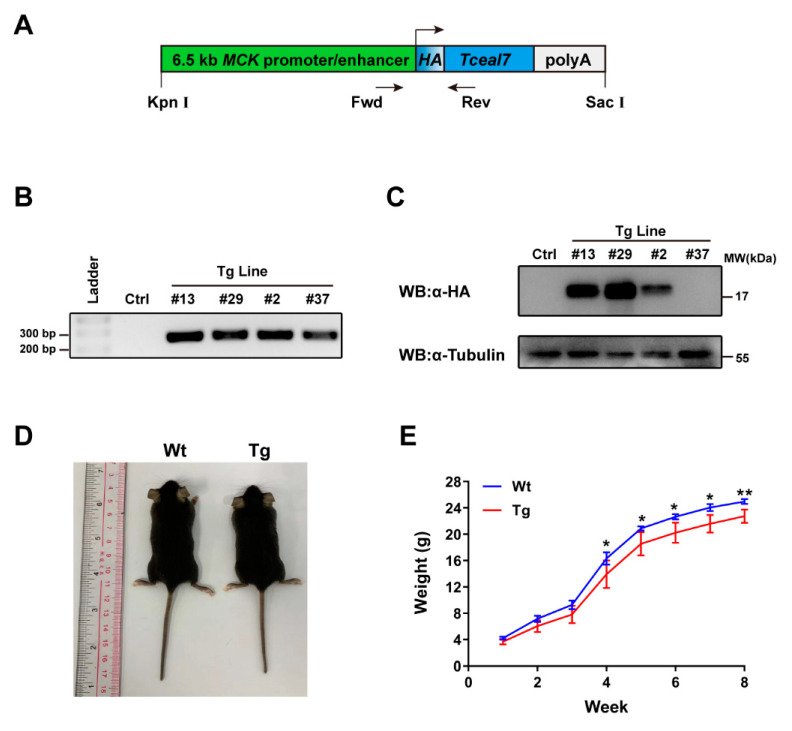
MCK 6.5 kb-HA-Tceal7 transgenic lines. (**A**) Schematic diagram of the MCK 6.5 kb-HA-Tceal7 transgenic vector. The transgenic fragment is isolated with the digestion of restriction enzyme Kpn I and Sac I. Fwd and Rev, primer pair for PCR identification of transgenic mice. (**B**) Four Tceal7 transgenic lines have been identified by routine PCR, referred to as #13, #29, #2 and #37. (**C**) Expression levels of HA-Tceal7 protein are confirmed by WB in hindlimb skeletal muscle of #13, #29, #2 and #37 transgenic mice lines. Tubulin, loading control in WB analysis. (**D**) Whole-body of Tceal7 transgenic mouse is a little smaller than that of its wild-type littermate at 4 weeks. (**E**) Growth curves of Tceal7 transgenic mice and their wild-type littermates at 1–8 weeks after birth (n = 6). All data represent the mean ± SD. *, *p* < 0.05, **, *p* < 0.01.

We have recently investigated the transcriptional regulation of *Tceal7* in skeletal muscle, which is driven by a triple complex consisting of three transcription factors, Mef2c, Creb1 and Myod [23]. We have also reported that overexpression of Tceal7 inhibits myoblasts proliferation and promotes myoblasts differentiation [22]. However, the molecular mechanism of the Tceal7 function still remains to be elucidated. Herein, we have constructed a transgenic mouse model specifically overexpressing HA-Tceal7 in skeletal muscle tissue to further explore the functional role of Tceal7 in vivo. As shown in Figure 1A, the Tceal7 coding region sequence with a HA tag is inserted behind the MCK promoter and enhancer. The purified transgene fragments have been microinjected into 200 fertilized eggs to generate MCK 6.5 kb-HA-Tceal7 transgenic mice. Four genetically stable transgenic lines have been established after screening and mating: #2, #13, #29 and #37 Tg lines (Figure 1B). Among these transgenic lines, both #13 and #29 display high expression levels of HA-Tceal7, #2 with a mild expression, while no expression has been detected in the #37 Tg line (Figure 1C). Therefore, #13 and #29 Tg lines are maintained for further research. Data from #29 Tg line are present as the similarity between these two lines unless otherwise stated. We have observed that transgenic mice are born without any obvious physical defects but are significantly smaller than wild-type littermates during development (Figure 1D). We then continuously tracked the body weight changes of transgenic and wild-type littermates for eight weeks. As shown in Figure 1E, there is a very subtle difference in body weight between the transgenic and wild-type mice during the first three weeks but a significant difference from the fourth week afterward. Taken together, these data suggest that sustained high expression of Teal7 represses body weight gain in neonatal mice.

### 2.2. Characterization of the Skeletal Muscle Fiber in Tceal7 Transgenic Mice

**Figure 2:**
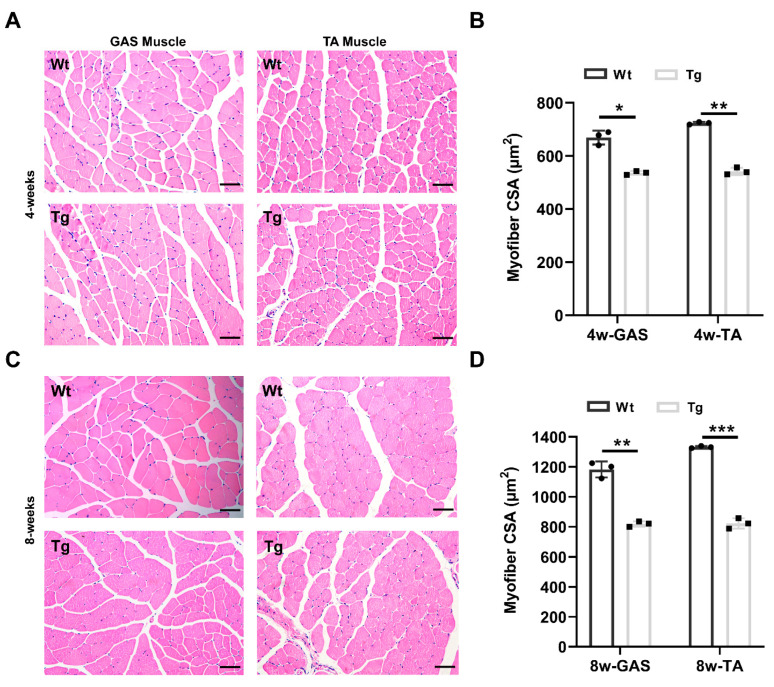
Characterization of skeletal muscle in MCK 6.5 kb HA-Tceal7 transgenic mice. (**A**) Hematoxylin and eosin staining of the gastrocnemius (GAS) and tibialis anterior (TA) muscles of 4-week transgenic mice and wild-type littermates. Scale bar = 50 μm. (**B**) Quantification of cross-sectional area (CSA) of myofibers presented in A from the gastrocnemius and tibialis anterior. *, *p* < 0.05; **, *p* < 0.01. (**C**) Hematoxylin and eosin staining of GAS and TA muscles of 8-week transgenic mice and wild-type littermates. Scale bar = 50 μm. (**D**) Quantification of CSA of myofibers in C from GAS and TA. **, *p* < 0.01; ***, *p <* 0.001. All data are the mean ± SD of three independent replicates.

Given that MCK 6.5 kb-HA-Tceal7 is specifically expressed in skeletal muscle lineage, we reason that the decreased body weight is due to a reduction of skeletal muscle growth, as skeletal muscle mass is about 40 percent of body weight. We have then examined the myofiber of the gastrocnemius and tibialis anterior muscles from four-week transgenic and wild-type littermates by hematoxylin and eosin staining. As shown in Figure 2A, there is no inflammation or denervation and other gross abnormalities observed in the H&E-stained muscle sections of the MCK 6.5 kb-HA-Tceal7 transgenic mice, but these myofibers are smaller than that of wild-type controls. The cross-sectional area of these myofibers is further quantitatively analyzed. As shown in Figure 2B, the average cross-sectional area of myofiber is decreased in both gastrocnemius and tibialis anterior of transgenic mice at the age of four weeks. Moreover, we have also examined myofiber morphology in eight-week transgenic and wild-type mice utilizing the same strategy. Interestingly, the average cross-sectional area of muscle fibers in gastrocnemius and tibialis anterior is further reduced in transgenic mice at the age of eight weeks compared with wild-type mice (Figure 2C,D). We have clearly observed myofiber growth in gastrocnemius and tibialis anterior muscle between four weeks and eight weeks, indicating that skeletal muscle is growing at a slower rate in transgenic mice (Figure 2B,D). As the skeletal muscle mass accounts for around 40% of body weight, the normalized weight difference of GAS and TA between transgenic mice and wild type littermates is not significant at four weeks or eight weeks (Figure S1). Overall, the above results demonstrate that overexpression of Tceal7 represses skeletal muscle myofiber growth in mice.

### 2.3. Tceal7 Directly Interacts with Cdk1

**Figure 3:**
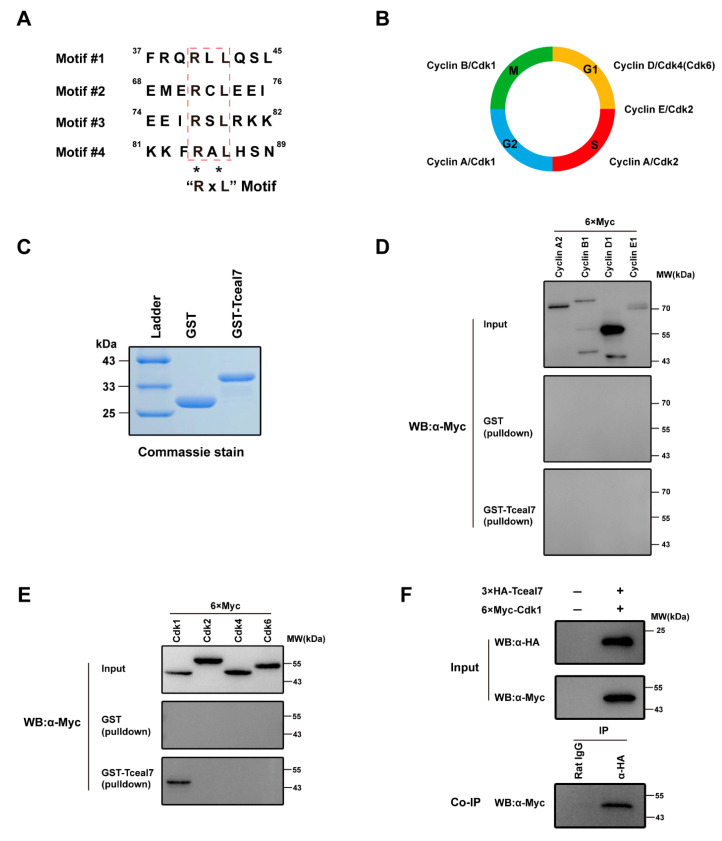
Direct interaction between Tceal7 and Cdk1. (**A**) Analysis of Tceal7 amino acid sequence. Four RxL motifs (Motif #1-#4) are marked by red dotted lines. (**B**) Schematic illustration of mammalian cell cycle regulation. The cell cycle consists of four phases: G1, S (DNA synthesis), G2 and M (mitotic). (**C**) GST and GST-Tceal7 fusion proteins have been purified by gel beads and analyzed by Coomassie brilliant blue staining. (**D**) Expression of cyclins (Cyclin A2, Cyclin B1, Cyclin D1 or Cyclin E1) has been analyzed by Western blot (WB) with a Myc antibody (Upper panel). The pulldown complex has then been analyzed by western blot (Middle and Lower panels). None of these cyclin proteins can interact with Tceal7. (**E**) Expression of Cdks (Cdk1, Cdk2, Cdk4 or Cdk6) has been verified by WB (Upper panel). Cdk1 is the only Tceal7-binding Cdk instead of Cdk2, Cdk4 or Cdk6 (Middle and Lower panel). (**F**) Expression of 3×HA-Tceal7 and 6×Myc-Cdk1 has been confirmed by WB. 6xMyc-Cdk1 has been detected in the co-immunoprecipitation complex by an HA antibody, but not Rat IgG control.

Cell cycle exit is a pivotal step in myogenic differentiation, which is regulated by multiple players, including Cyclin, Cdk, Rb1, E2F1, DP1, p21, etc. [24,25,26,27]. Previous studies have shown an inhibitory function of Tceal7 in myoblast proliferation [22], while the above data have demonstrated its repression function in skeletal muscle growth. Bioinformatics analysis has revealed four RxL motifs in the 98 amino acid residues of Tceal7 (Figure 3A), which may mediate its interaction with Cyclin proteins [1]. Cyclin/Cdk complexes have complicated roles in both cell cycle progression and skeletal muscle growth [1,5,8]. Cell cycle progression is driven by the orderly organized Cdk/Cyclin complexes, such as Cyclin D/Cdk4(Cdk6), Cyclin E/Cdk2, Cyclin A/Cdk2, Cyclin A/Cdk1, and Cyclin B/Cdk1 (Figure 3B) [1].

These data prompt us to examine the relationship between Tceal7 and Cdk/Cyclin complexes. Therefore, we have first investigated the protein-protein interaction between Tceal7 and these Cyclins. GST and GST-Tceal7 proteins have been overexpressed in BL21 *E. coli* and then purified with GST affinity beads for GST-pulldown analysis (Figure 3C). As shown in Figure 3D, GST-Tceal7 could not pulldown any cyclin, including Cyclin A2, Cyclin B1, Cyclin D1 and Cyclin E1, suggesting that there is no direct association between Tceal7 and cyclin proteins. We reason that the additional sequences surrounding these four RxL motifs are required for the interaction between Tceal7 and Cyclin proteins [1,2]. We have then assessed the binding of Tceal7 to diverse Cdks utilizing a similar strategy. Surprisingly, Tceal7 is specifically associated with Cdk1, rather than Cdk2, Cdk4 or Cdk6 (Figure 3E). In order to further confirm their interaction in vivo, we have overexpressed Tceal7 and Cdk1 together in HEK293T cells for co-immunoprecipitation experiments. As shown in Figure 3F, 6×Myc-Cdk1 is co-immunoprecipitated with 3xHA-Tceal7 by a HA antibody, but not the normal Rat IgG control, which supports the notion that Tceal7 interacts with Cdk1 in vivo.

### 2.4. Identification of the Interacting Domains between Tceal7 and Cdk1

**Figure 4:**
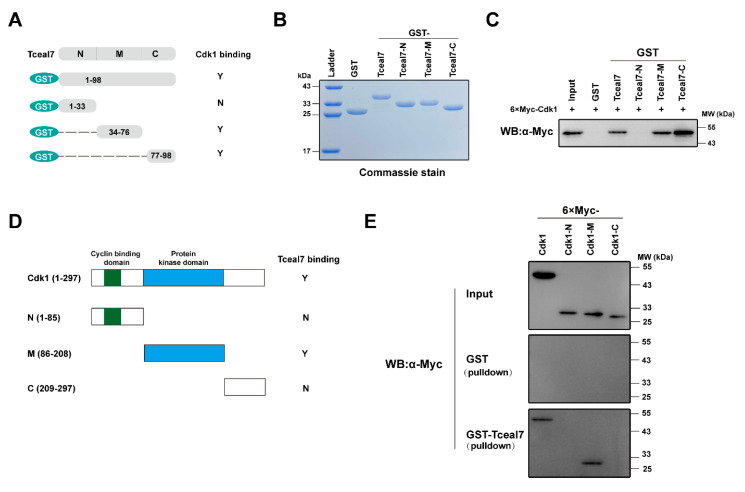
Protein-protein interaction between Tceal7 and Cdk1. (**A**) Schematic diagram of the interaction between Tceal7 deletional mutants and Cdk1. Y, yes; N, no. (**B**) GST, GST-Tceal7 and its deletional mutant fusion proteins have been purified and analyzed by Coomassie blue staining. (**C**) 6×Myc-Cdk1 can be pulled down by GST-Tceal7 (1–98), GST-Tceal7-M (34–76) and GST-Tceal7-C (77–98), but not GST-Tceal7-N (1–33). (**D**) Schematic summary of the interaction between Cdk1 deletional mutants and Tceal7. Cyclin binding domain, which is responsible for binding to cyclin through the αC-helix (PSTAIREISLL); Protein kinase domain, which is involved in the phosphorylation of specific substrate. Y, yes; N, no. (**E**) Expression of 6×Myc-Cdk1 and its deletions have been examined with an anti-Myc antibody (Upper panel). Both 6×Myc-Cdk1 (1–297) and 6×Myc-Cdk1-M (86–208) can be pulled down by GST-Tceal7, but not 6×Myc-Cdk1-N (86–208) or 6×Myc-Cdk1-C (209–297) (Middle and Lower panel).

Identification of Cdk1 as a novel associated protein of Tceal7 prompts us to further characterize the protein-protein interaction between Tceal7 and Cdk1. We have then constructed a series of deletional mutants of Tceal7 and Cdk1 for GST-pulldown assays. Tceal7 deletional constructs and their interactions with Cdk1 are summarized in Figure 4A. GST, GST-Tceal7(1–98), GST-Tceal7-N (1–33), GST-Tceal7-M (34–76) and GST-Tceal7 (77–98) have been purified from *BL21 E. coli* and verified by Coomassie brilliant blue staining (Figure 4B). Interestingly, in addition to the full length of Tceal7 (1–98), both Tceal7-M (34–76) and Tceal7-C (77–98) can interact with Cdk1, while Tceal7-N (1–33) does not have the ability (Figure 4C). Therefore, it is possible that there are two Cdk1-binding motifs in the region (aa34–98) of Tceal7. We have then assessed the domain(s) of Cdk1 that mediates its association with Tceal7 in a similar strategy, which is summarized in Figure 4D. As shown in Figure 4E, each deletional Cdk1 protein is expressed in HEK293T cells with a lower level than that of the full-length (upper panel). Further GST-pulldown assays reveal that GST-Tceal7 (1–98) is able to pull down Cdk1 full length and Cdk1-M (86–208), but not Cdk1-N (1–85) or Cdk1-C (209–297) (Figure 4E, middle and lower panel). Collectively, these data suggest that the protein-protein interaction between Tceal7 and Cdk1 is mediated through the region (aa34–98) of Tcea7 and the M domain (aa86–208) of Cdk1.

### 2.5. Reduction of Protein Phosphorylation in MCK 6.5 kb HA-Tceal7 Transgenic Mice

**Figure 5:**
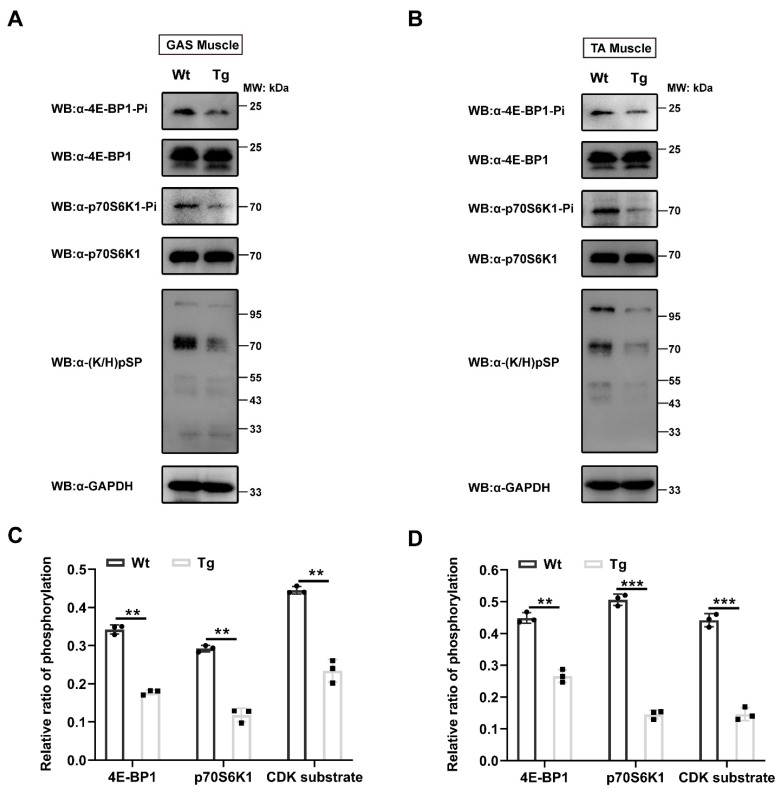
Protein phosphorylation in the skeletal muscle of Tceal7 transgenic mice. (**A**,**B**) The phosphorylation levels of 4E-BP1, p70S6K1 and Cdk substrates in the gastrocnemius (**A**) and tibialis anterior (**B**) muscles of 8-week-old transgenic and wild-type littermates have been analyzed by Western Blot with specific antibodies. Total protein level of 4E-BP1 and p70S6K1 has been analyzed with α-4E-BP1 and α-p70S6K1, respectively. α-GAPDH, protein loading control. (**C**,**D**) Relative ratios of phosphorylation of 4E-BP1 Ser65, p70S6K1 Thr389 and CDK-pS in gastrocnemius (**C**) and tibialis anterior muscles (**D**). Note that the relative ratios of phosphorylation are quantified as [4E-BP1 pSer65]/[4E-BP1], and [p70S6K1 pThr389]/[p70S6K1], and [Cdk substrate (K/H)pSP]/[GAPDH]. All data represent the mean ±SD of three biologically independent samples. **, *p <* 0.01; ***, *p <* 0.001.

Our above studies have revealed that overexpression of Tceal7 results in the reduction of skeletal muscle fiber size and that Tceal7 binds to Cdk1 specifically. Accumulating evidence has shown that the activity of Cdk1 is also critical for protein synthesis through its downstream targets 4E-BP1 and p70S6K1 during skeletal muscle development [9,28,29,30]. Our above studies indicate that the protein-protein interaction between Tceal7 and Cdk1 may regulate the kinase activity of Cdk1. Therefore, we have examined the protein phosphorylation of 4E-BP1, p70S6K1 and Cdk1 substrates in the skeletal muscle of MCK 6.5 kb HA-Tceal7 transgenic mice. As shown in Figure 5A,B, the phosphorylation of 4E-BP1 Ser65 and p70S6K1 Thr389 is downregulated in both gastrocnemius and tibialis anterior muscles from transgenic mice compared with wild-type control, while the protein expression of 4E-BP1 and p70S6K1 is not altered in the transgenic mice. Further quantitative assays have revealed that the relative ratios of phosphorylation of 4E-BP1 Ser65 and p70S6K1 Thr389 in the gastrocnemius or tibialis anterior muscle decreased from 0.34 to 0.18 and from 0.30 to 0.12, or from 0.45 to 0.27 and from 0.51 to 0.14 in transgenic mice (Figure 5C,D). These results indicate that Tceal7 may reduce the phosphorylation levels of 4E-BP1 Ser65 and p70S6K1 Thr389 by inhibiting the activity of Cdk1. To further ascertain this possibility, we have evaluated the phosphorylation of Cdk substrates in the gastrocnemius and tibialis anterior muscles of transgenic mice. As expected, the relative ratios of phosphorylation of Cdk substrates have been attenuated in both gastrocnemius (from 0.45 to 0.23) and tibialis anterior muscles (from 0.44 to 0.15) in transgenic mice (Figure 5). In summary, the phosphorylation of 4E-BP1 Ser65, p70S6K1 Thr389 and Cdk substrates have been downregulated in the skeletal muscle of MCK 6.5 kb HA-Tceal7 transgenic mice.

## 3. Discussion

**Figure 6:**
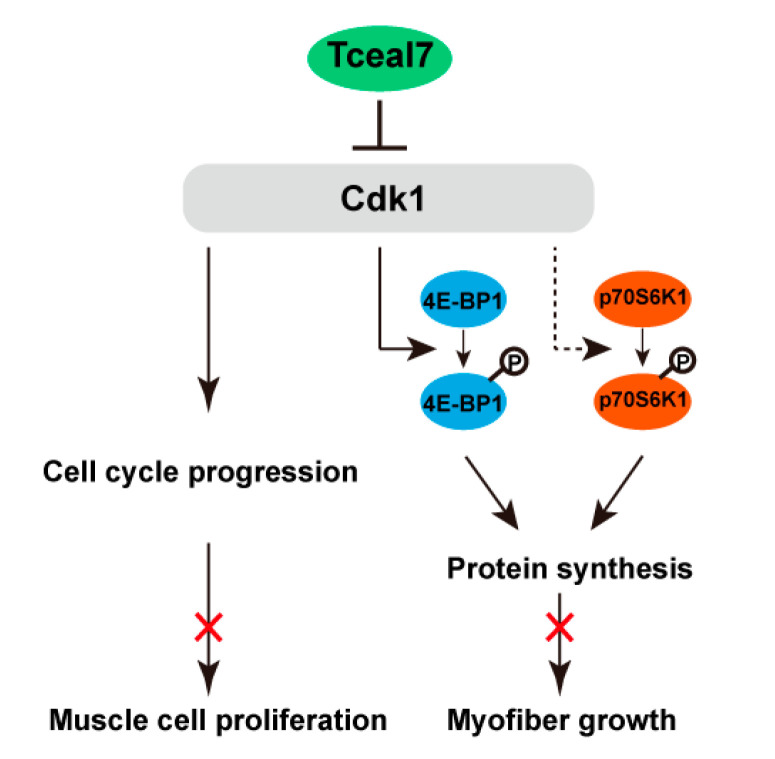
The regulatory mechanism of Tceal7 in skeletal muscle development. Tceal7 interacts with Cdk1 and subsequently suppresses the activity of the Cdk1, resulting in attenuated muscle cell proliferation (Lower left). The decreased activity of Cdk1 also results in the lower phosphorylation level of 4E-BP1 (directly, arrow) and p70S6K1 (indirectly, arrowhead with dotted line), which leads to a decrease in protein synthesis and ultimately retarded myofiber growth (Lower right).

Expression of Tceal7 has presented a very interesting pattern during skeletal muscle development and regeneration [22]. We have recently reported that CRE#3 in its 0.7 kb promoter may be the critical element for this dynamic character [23]. However, the functional significance of this pattern is still mysterious, although overexpression of Tceal7 in myoblast cells attenuates their proliferation and promotes myogenic differentiation. In the present study, a regulatory 6.5 kb fragment from the *MCK* gene is utilized to construct the MCK 6.5 kb HA-Tceal7 transgenic model, in which a HA tag is fused to the *Tceal7* gene due to the unavailability of Tceal7 antibody. Our data support a novel model that Tceal7 represses Cdk1 activity via its inhibitory interaction with Cdk1, thereby repressing muscle cell proliferation and myofiber mass accumulation (Figure 6).

First, we have discovered that Tceal7 also serves as a negative regulator of skeletal muscle development during postnatal development, while our previous studies have demonstrated a positive regulatory role of Tceal7 in myogenic differentiation [22]. This unique character of Tceal7 has distinguished itself from other regulatory genes, such as *Igf1* and *Tgr5* [31,32,33,34,35,36]. Igf1 promotes myogenic differentiation through the PI3K/Akt signaling or myostatin signaling, and overexpression of Igf1 results in a hypertrophy phenotype via mTOR or GSK3β signaling [31,32,33,34,35]. Overexpression of Tgr5 enhances C2C12 myogenic differentiation, and transgenic overexpression of Tgr5 driven by MCK promoter and enhancer induces skeletal muscle hypertrophy [36]. These dual roles of Tceal7 in skeletal muscle development provide a satisfactory explanation of Tceal7 dynamic expression: Tceal7 is highly expressed upon the early stage of myogenic differentiation as Tceal7 promotes myoblast cell cycle exit and then down-regulated as Tceal7 represses mass accumulation in the mature muscle fiber.

The identification of Cdk1 as an interacting protein of Tceal7 is the second discovery in the present study. Initially, we hypothesize that Tceal7 can bind the cyclin proteins, such as Cyclin A2, Cyclin B1, Cyclin D1 and Cyclin E1, as four RxL motifs have been identified in the Tcea7 protein sequence. However, in vitro binding experiments could not support this hypothesis. Intensive research has revealed that the neighbor sequences of the RxL motif are also required for its interaction with cyclin proteins, as the consensus RxL motif is defined as R/K-x-L-φ or R/K-x-L-x–φ [1,2,37,38,39,40], which may be the reason of our observation. Our following analysis has identified Cdk1 as a Tceal7-binding protein. Surprisingly, we have not observed the interaction between Tceal7 and Cdk2, although the protein sequences of Cdk1 and Cdk2 are highly homologous [41,42]. The underlying mechanism remains to be defined in future research. Deletional studies have revealed the characterization of the interaction between Cdk1 and each part of Tceal7. As the interaction between C-terminal and Cdk1 is stronger than that of full-length, the N-terminal may inhibit the interaction between Tceal7 and Cdk1 through a mechanism unknown yet. Mapping studies have also shown that Tceal7 specifically binds to Cdk1 kinase domain M (86–208) instead of N (1–85) or C (209–297) domains, which prompted us to examine the hypothesis that Tceal7 may modulate the activity of Cdk1.

The demonstration of inhibiting Cdk1 activity by Tceal7 is our third finding here. Our previous studies have shown that overexpression of Tceal7 slows down myoblast proliferation and promotes myogenic differentiation [22]. Herein, our data have revealed that Tceal7 represses the phosphorylation of Cdk1 substrates, thereby inhibiting myoblasts proliferation. Myogenic differentiation is a highly integrated process: irreversible exit from cell cycle progression, muscle differentiation characterized by the transactivation of muscle-specific genes, and formation of multinucleated myotube [43,44]. Therefore, enhancement of myogenic differentiation by Tceal7 may be an indirect outcome of inhibition of cell proliferation. Given the complexity of skeletal muscle development, Tceal7 may also directly regulate muscle differentiation, which remains to be investigated in the future. Cdk1 has also been reported as a substitute protein kinase of mTOR for phosphorylation of 4E-BP1 Thr70, Ser65/Ser101, and Thr37/Thr46 to activate mRNA translation [28]. Overexpression of 4E-BP1 with mTOR phosphorylation site mutation retards skeletal muscle cell growth, while there is no skeletal muscle defect in the double knockout of 4E-BP1 and 4E-BP2 mice [45,46]. In line with these findings, we have observed a reduction of phosphorylation of 4E-BP1 Ser65 and smaller myofibers in the adult skeletal muscle from MCK 6.5 Kb HA-Tceal7 transgenic mice. It will strengthen our conclusion to examine the regulation of Cdk1 activity by Tceal7 through in vitro experiments in the future.

Interestingly, it has been reported that Cdk1 represses the phosphorylation of p70S6K1 Thr389 indirectly instead of enhancing its phosphorylation in a transformed cell line [30]. However, in vivo studies have shown that deletion of p70S6K1 in mice causes the reduction of body weight and skeletal muscle mass, accompanied by a decrease in the cross-sectional area of the myofibers, while myotubes overexpressing p70S6K1 (a constitutively active form) induces muscle hypertrophy [47,48]. Consistent with these studies from mice, we have observed reduced phosphorylation of p70S6K1 Thr389 upon inhibition of Cdk1 activity in the Tceal7 transgenic skeletal muscle. Therefore, our present work supports the notion that Cdk1 may also regulate the phosphorylation of p70S6K1 Thr389 positively through a mechanism unknown yet. The discrepancy may be due to the difference between the mitotic cell line and postmitotic skeletal muscle fiber. Recent reports have highlighted that high expression of Cdk1 is correlated with the growth of cancers and poor prognoses, such as epithelial ovarian cancer, breast cancer and lung cancer [49,50,51]. Reducing Cdk1 expression and inhibiting Cdk1 activity have become effective therapeutic approaches [52].

Early studies have shown that human TCEAL7 can inhibit Cyclin D1 expression induced by c-Myc in ovarian epithelial cells and negatively regulate NF-κB signaling through repressing p65 transcriptional activity in ovarian cancer cells [53,54]. A recent report has revealed that TCEAL7 regulates epithelial-mesenchymal transition, invasion and metastasis of breast cancer through the NF-κB pathway [55]. An additional report has also demonstrated that TCEAL7 interacts with β-catenin and prevents its nuclear translocation, thereby obstructing Wnt/β-catenin signaling in glioblastoma [56]. These studies have supported the idea that human TCEAL7 is a negative regulator of cell proliferation, which is consistent with our findings.

Taken together, we have discovered a novel role of Tceal7 in skeletal muscle development through its inhibitory interaction with Cdk1. Human TCEAL7 has presented a 76% homology and 89% similarity to mouse Tceal7 regarding to protein sequence. Therefore, it is worthy to further investigate in the future whether human TCEAL7 regulates Cdk1 activity in cancer progression through the mechanism revealed in our present work, which may provide a novel strategy for cancer treatment.

## 4. Materials and Methods

### 4.1. Plasmid Construction

Myotube RNA extraction and subsequent cDNA synthesis were performed as previously described utilizing RNA Extraction Kit and cDNA Synthesis Kit (Aidlab Biotechnologies, Beijing, China) [23]. MCK 6.5 kb-HA-Tceal7 transgenic vector was constructed through two steps: firstly, the coding region of *Tceal7* (NM_001127169.1) was cloned into pcDNA-HA expression vector through routine PCR; Secondly, the *HA-Tceal7* fragment digested with Pst I and Xba I from pcDAN-HA-Tceal7 plasmid was subcloned to pBSK MCK promoter vector (Addgene plasmid #12528, Watertown, MA, USA). The following genes were all subcloned to pCS2-6×Myc expression vector through PCR: *Ccna2* (NM_009828.3), *Ccnb1* (NM_172301.3), *Ccnd1* (NM_007631.3), *Ccne1* (NM_007633.2), *Cdk1* (NM_007659.4), *Cdk2* (NM_183417.3), *Cdk4* (NM_009870.4), *Cdk6* (NM_009873.3) and deletional mutants of Cdk1. Moreover, *Tceal7* and its deletional mutants were also subcloned to the pGEX-4T-1 vector by PCR. All of these plasmids have been verified through DNA sequencing.

### 4.2. Cell Culture and Cell Transfection

HEK293T cells were cultured in high-glucose Dulbecco’s Modified Eagle Medium (DMEM) (Hyclone, Logan, UT, USA) supplemented with 10% FBS (Hyclone, Logan, UT, USA), 100 U/mL penicillin, 100 μg/mL streptomycin, and 2 mM-Glutamine. All cells were incubated in a 5% CO_2_ incubator at 37 °C. HEK293T cells transfection was carried out according to the manufacturer’s instructions of TransIntroTM EL Transfection Reagent (Transgen, Biotech, Beijing, China). Briefly, HEK293T cells were seeded into a 6-cm petri dish 24 h before transfection. On the next day, DNA and transfection reagent was added to an appropriate volume of opti-MEM serum-free medium (Sigma-Aldrich, St. Louis, MO, USA) in a ratio of 1:3, incubated at room temperature for 15 min, and then transferred to the HEK293T cells.

### 4.3. Coimmunoprecipitation Assays and Western Blot Analysis

The coimmunoprecipitation assays and western blot were performed as previously reported [57]. Briefly, HEK293T cells were co-transfected with 3×HA-Tceal7 and 6×Myc-Cdk1 expression plasmids for 24 h, washed twice with sterile PBS, and then lysed. The total lysate supernatant was aliquot and incubated with an anti-HA antibody with a dilution of 1:50 (Clone BMG-3F10, Roche, Switzerland) or a Rat IgG control (sc-2006, Santa Cruz Biotechnology, Santa Cruz, CA, USA) for overnight at 4 °C. The next day, each reaction was supplied with 40 μL protein A/G agarose for another 2 h at 4 °C. The agarose beads were washed thrice and resuspended in 30 μL 2 × SDS loading buffer. The immunoprecipitation complex was analyzed by western blot with an anti-Myc antibody with a dilution of 1:1000 (#2272, Cell Signaling Technology, Danvers, MA, USA).

### 4.4. GST-Pulldown Assays

GST, GST-Tceal7 and GST-Tceal7 deletions were overexpressed in BL21 *E. coli* induced with 0.1 mM IPTG for 3 h at 200 rpm at 37 °C. These bacteria were collected and lysed in a bacterial active protein extraction reagent (Beyotime, Beijing, China) supplemented with protease inhibitor for 15 min at room temperature. The lysate supernatant was separated by centrifugation at 13,000× *g* at 4 °C for 10 min and then incubated with 50 μL GST-tag purification resin for 30 min at 4 °C. The beads conjugated with GST or GST fusion protein were collected and washed thrice with precooled PBS containing 0.1% Triton X-100, followed by resuspension in sterile PBS. The purified proteins were examined by Coomassie brilliant blue staining and then quantitated through a BCA kit (Leagene Biotechnology, Beijing, China). For GST pulldown assays, 10 μg GST-Tceal7 or its deletions bound to beads were incubated with a cell lysate overexpressing 6×Myc tagged Cyclin or Cdk protein for 2 h at 4 °C. The precipitation was washed thrice with a precooled binding buffer (PBS supplemented with 10% glycerol and 1% Triton X-100) and then resuspended in 30 μL 2 × SDS loading buffer. Pulldown with GST protein was performed simultaneously as a control. The final pulldown proteins were analyzed by western blot with an anti-Myc antibody (1:1000 dilution).

### 4.5. Transgenic Mouse Lines Establishment and Animal Care

The pBSK MCK 6.5 kb promoter-HA-Tceal7 transgenic plasmid was digested with Kpn I and Sac I to obtain a linear 7 kb DNA fragment for microinjection. The purified transgene DNA was microinjected into 200 oocytes and then transplanted into pseudopregnant female mice, which was entrusted to GemPharmatech (Nanjing, China). To identify transgenic lines from newborn neonates, a genotyping was performed utilizing a pair of specific primers (Forward 5′-CAACTTGGGCTCCTGATGTT-3′ and Reverse 5′-CCATGGATCCAGCGTAATCT-3′). A total of 20 transgenic mice were identified, of which six males (#2, #13, #29, #37, #40 and #65) were mated with wild-type females to generate F1 generation mice. The #40 transgenic line failed to impregnate wild-type females. F1 pups from the #65 transgenic line were all negative. All of the remaining transgenic lines (#2, #13, #29, #37) were stably inherited, and the expression of transgene HA-Tceal7 was analyzed in their hindlimb muscles with an anti-HA antibody with a dilution of 1:1000 (Clone BMG-3F10, Roche, Switzerland) and anti-Tubulin antibody with a dilution of 1:1000 (sc-8035, Santa Cruz Biotechnology, Santa Cruz, CA, USA). Transgenic lines #13 and #29 were maintained as two Founder lines for further analysis. All mice used in this study had a C57BL/6J genetic background and were maintained at the Experimental Animal Center of the South China University of Technology. The mice were anesthetized by CO_2_ inhalation and euthanized by cervical dislocation under anesthesia. All animal experiment protocols were approved by the Review Committee of the Academy of Life Sciences, South China University of Technology, in 2018 (approval number 2018044), in compliance with ethical regulations and institutional guidelines.

### 4.6. Detection of Protein Phosphorylation

The gastrocnemius and tibialis anterior muscles of eight-week-old mice were isolated, minced, and then homogenized in an NP40 Buffer supplied with protease inhibitors and phosphatase inhibitors (Leagene Biotechnology, Beijing, China). These protein lysates were then quantified and analyzed by western blot with antibodies as follows: phospho-4E-BP1 antibody (#9451, Cell Signaling Technology, Danvers, MA, USA), phospho-p70 S6 kinase antibody (#9234, Cell Signaling Technology, Danvers, MA, USA), phospho-CDK Substrate Motif antibody (#9477, Cell Signaling Technology, Danvers, MA, USA), 4E-BP1 antibody (#9644, Cell Signaling Technology, Danvers, MA, USA), p70 S6 kinase antibody (#2708, Cell Signaling Technology, Danvers, MA, USA) and GAPDH antibody (#A19056, ABclonal Technology, Wuhan, China). The dilution ratio of these antibodies for detecting phosphorylation was 1:500, while that of all other antibodies was 1:1000. The signal was detected in a chemiluminescence ChemiScope 4300Pro system and quantified with Clinx Image Analysis software.

### 4.7. Hematoxylin and Eosin Staining

Hematoxylin and eosin staining was carried out as previously characterized [58]. The gastrocnemius and tibialis anterior muscles of four-week-old and eight-week-old mice were isolated and fixed with 4% paraformaldehyde overnight at 4 °C before sectioning. The tissue sections were deparaffinized, hydrated, stained and air-dried prior to imaging under a microscope. The cross-sectional area of myofibers was quantified using ImageJ software and calculated for at least 300 myofibers in each group.

### 4.8. Statistics

All data represent the means of at least three independent replicates. Statistical significance was performed by Student’s t-test for two group comparisons and one-way ANOVA for multiple comparisons with a Tukey’s test using a GraphPad Prism. *, *p* < 0.05; **, *p* < 0.01; and ***, *p* < 0.001.

## 5. Conclusions

In summary, our data fully support the notion that Tceal7 represses myoblast proliferation and myofiber growth through its inhibitory interaction with Cdk1. The exhibited function of Tceal7 corresponds almost exactly to its spatiotemporal expression pattern, with high expression during embryonic development or skeletal muscle regeneration but reverting to basal level after birth or completion of impaired regeneration.

## Data Availability

Not applicable.

## Supplementary Materials

The following supporting information can be downloaded at: https://www.mdpi.com/article/10.3390/ijms24076264/s1.
