# Supplementary material for: Tceal7 Regulates Skeletal Muscle Development through Its Interaction with Cdk1
Source: Int J Mol Sci. 2023 Mar 27;24(7):6264. doi: 10.3390/ijms24076264 (PMC10094454; doi:10.3390/ijms24076264)
Supplement: Supplementary file 1 [file ijms-24-06264-s001.zip › ijms-2282601-supplementary.pdf]

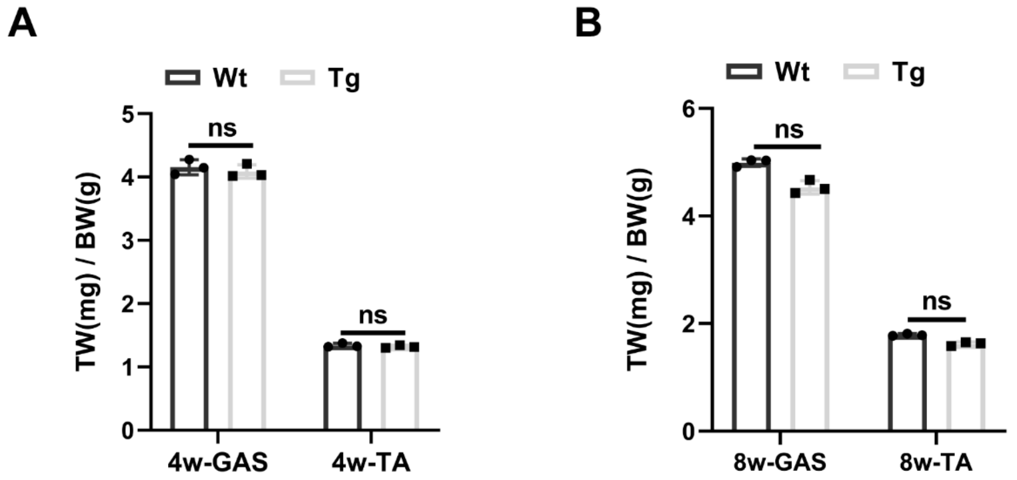

Figure S1. Normalized muscle weight of MCK 6.5 kb-HA-Tceal7 transgenic mice and wild-type littermates. The weights of GAS and TA muscles are normalized to total body weight of mice at the age of 4-weeks (A) and 8-weeks (B). All data represent the mean ± SD of three independent samples. ns, not significant. TW, tissue weight; BW, body weight.
